# Electrochemically Activated Conductive Ni-Based MOFs for Non-enzymatic Sensors Toward Long-Term Glucose Monitoring

**DOI:** 10.3389/fchem.2020.602752

**Published:** 2020-11-25

**Authors:** Yating Chen, Yulan Tian, Ping Zhu, Liping Du, Wei Chen, Chunsheng Wu

**Affiliations:** Department of Biophysics, School of Basic Medical Sciences, Health Science Center, Xi'an Jiaotong University, Xi'an, China

**Keywords:** electrochemical, conductive Ni-MOFs, cyclic voltammetry, non-enzymatic, glucose sensor

## Abstract

Continuous intensive monitoring of glucose is one of the most important approaches in recovering the quality of life of diabetic patients. One challenge for electrochemical enzymatic glucose sensors is their short lifespan for continuous glucose monitoring. Therefore, it is of great significance to develop non-enzymatic glucose sensors as an alternative approach for long-term glucose monitoring. This study presented a highly sensitive and selective electrochemical non-enzymatic glucose sensor using the electrochemically activated conductive Ni_3_(2,3,6,7,10,11-hexaiminotriphenylene)_2_ MOFs as sensing materials. The morphology and structure of the MOFs were investigated by scanning SEM and FTIR, respectively. The performance of the activated electrode toward the electrooxidation of glucose in alkaline solution was evaluated with cyclic voltammetry technology in the potential range from 0.2 V to 0.6 V. The electrochemical activated Ni-MOFs exhibited obvious anodic (0.46 V) and cathodic peaks (0.37 V) in the 0.1 M NaOH solution due to the Ni(II)/Ni(III) transfer. A linear relationship between the glucose concentrations (ranging from 0 to 10 mM) and anodic peak currents with R2 = 0.954 was obtained. It was found that the diffusion of glucose was the limiting step in the electrochemical reaction. The sensor exhibited good selectivity toward glucose in the presence of 10-folds uric acid and ascorbic acid. Moreover, this sensor showed good long-term stability for continuous glucose monitoring. The good selectivity, stability, and rapid response of this sensor suggests that it could have potential applications in long-term non-enzymatic blood glucose monitoring.

## Introduction

Diabetes, caused by insulin deficiency or resistance, is a chronic disorder of glucose metabolism. It has become one of the most widespread diseases in the world and has profound implications for disability and mortality (Wong et al., 2013). According to the World Health Organization, it is estimated that type II diabetes patients will grow to at least 350 million worldwide by 2030 if no appropriate action is taken (Collins et al., 2011). The normal range of blood glucose concentration is 4.4-6.6 mM, while the blood glucose concentration in patients with diabetes mellitus is out of this normal range (Chelaghmia et al., 2018). To care for diabetes, the blood glucose level of patients needs to be monitored on a regular basis daily. Thus, establishing a fast and reliable estimation method to detect blood glucose concentrations has attracted considerable interest from scientists.

Currently, an electrochemical enzyme-based glucose biosensor has been used widely because of its high selectivity, reliability, and simplicity (Caliò et al., 2016; Karimi-Maleh et al., 2020a). However, the instability, critical operating conditions, and limited lifetimes of enzymes hinder the application of this biosensor for continuous glucose monitoring (Zhang et al., 2013). To address these drawbacks, non-enzymatic electrochemical glucose biosensors, including noble metallic materials and their alloys, have been proposed and developed (Dolinska et al., 2014; Chang et al., 2015; Shen et al., 2015; Ye et al., 2015; Wei et al., 2018; Karimi-Maleh et al., 2020b). However, the cost of noble metals was high. Recently, it was found that some transition metals and their metal oxides or hydroxides, such as Cu, Co, Ni, Cu(OH)_2_, Co(OH)_2_, NiO, Ni(OH)_2_, NiS_2_, and ZnO, have exhibited non-enzymatic glucose sensing performance (Song et al., 2015; Wei et al., 2015; Darvishi et al., 2017; Du et al., 2019; Hayat et al., 2019; Sun et al., 2019; Padideh et al., 2020; Shi et al., 2020). Among these, nickel-based sensors show excellent electrochemical activity toward glucose oxidation (Martins et al., 2011; Gao et al., 2016).

Metal-organic frameworks (MOFs), which are constituted by organic linkers and metal ions or clusters, have rapidly been developed (Jiao et al., 2019). MOFs are a new type of crystalline material with ultrahigh surface areas up to 10,000 m^2^/g, high surface area to volume ratios, tunable structures, and flexible tailorability. They have been applied in various fields including gas sorption and separation, catalysis, sensors, cancer therapy, and drug delivery (Li et al., 2009; Lu et al., 2018; Zhou et al., 2018; Liang et al., 2019; Rojas et al., 2019; Wang et al., 2020). For electrochemical glucose sensors, Cu-MOFs, Co-MOFs, Ni-MOFs, and their metal oxide composites have been developed (Sun et al., 2018; Li et al., 2020b; Liu et al., 2020; Qiao et al., 2020; Shahrokhian et al., 2020). Conductive Ni_3_(2,3,6,7,10,11-hexaiminotriphenylene)_2_ (2,3,6,7,10,11-hexaiminotriphenylene=HITP) MOFs exhibit very high electrical conductivity, exceeding those of previous semiconducting metal-organic graphene analogs and other conductive MOFs, which are even higher than some of the best organic conductors (Sheberla et al., 2014). Thus, it is suspected that the high porosity, high surface area, large pore diameter, and high conductivity of these MOFs could promote the electron transfer between the electrode and solution.

In this study, the electrochemically activated conductive Ni_3_(2,3,6,7,10,11-hexaiminotriphenylene)_2_ MOFs was utilized as sensing materials to develop electrochemical non-enzymatic glucose sensors. The morphology and structure of MOFs were characterized by scanning SEM and FTIR, respectively. In fact, after the activation of Ni-MOFs in KOH solution, the conductive MOFs exhibited stable reduction and oxidation peaks in alkaline solution, which was very sensitive to glucose concentrations. Moreover, the activated Ni-MOFs showed excellent selectivity and stability. All the obtained results suggest the promising prospects and potential applications of this sensor for non-enzymatic and long-term blood glucose monitoring.

## Experimental Section

### Materials

HITP·6HCl (Chemical Reagent) were brought from Shanghai TenSus Biotechnology Co., Ltd. NiCl_2_·6H_2_O (Analytical Reagent) and Nafion (5% solution) was brought from Sigma-Aldrich. Potassium hexacyanoferrate (K_4_Fe(CN)_6_), triethylamine, glucose, KCl, KOH, uric acid (UA), and ascorbic acid (AA) of an analytical reagent were purchased from Sinopharm Chemical Reagent Co., Ltd. All chemicals were of an analytical reagent grade and were used without further purification. All solutions were prepared by distilled water and stored at room temperature.

### Preparation of Ni_3_(HITP)_2_ MOFs

The preparation of Ni-MOFs followed previous reports with some modifications (Sheberla et al., 2014). Typically, a solution of 6.6 mg of NiCl_2_·6H_2_O in 5 mL of water and 0.1 mL of triethylamine solution was added to a solution of 10 mg of HITP·6HCl in 5 mL of water. The mixture was stirred under an oil bath at 65°C for 3 h. The resulting black powder was centrifuged, filtered, and washed with water several times and then dried under a vacuum oven for 24 h.

### Preparation of Modified Electrodes

Electrochemical experiments were performed on an electrochemical station (CHI600E). A standard three-electrode cell was applied in all the measurements. Ni-MOFs modified glass carbon electrode (GCE) and electrochemical treated Ni-MOFs electrode were used as working electrodes. The platinum rod and saturated calomel electrode (SCE) were used as counter electrodes and reference electrodes, respectively. All the electrochemical tests were performed at room temperature.

The GCE was polished with 1.5 μm, 0.5 μm, and 50 nm aluminum oxide sequentially until the P_anodic_-P_cathodic_ in 0.1 M K_4_Fe(CN)_6_/0.01M KCl below 80 mV vs. SCE. A mixture solution of 16 μL Ni-MOFs (1 mg mL^−1^) and 4 μL Nafion was added to the polished GCE surface and dried under room temperature. To active the Ni-MOFs, the cyclic voltammogram method was performed at the potential between 0.2 V to 0.6 V vs. SCE at a scan rate of 50 mV s^−1^ for 100 cycles in a 0.1 M KOH solution until stable curves were obtained.

### Electrochemical Measurements

The activated Ni-MOFs electrode was investigated as a glucose sensor in an alkaline medium. Cyclic voltammograms (CVs) measurements were processed in 0.1 M KOH in the potential range from 0.2 to 0.6 V with a scan rate of 50 mV s^−1^. The selectivity testes were applied in the mixture glucose with 10-fold concentration of AA and UA.

### Characterization

The morphology was observed by a scanning electron microscope (SEM, FEI Nova NanoSEM 450). The functional groups were tested by Fourier Transform Infrared Spectrometer (FTIR, Nicolet iS50).

## Results and Discussion

### Characterization of Ni-MOFs

**Figure 1:**
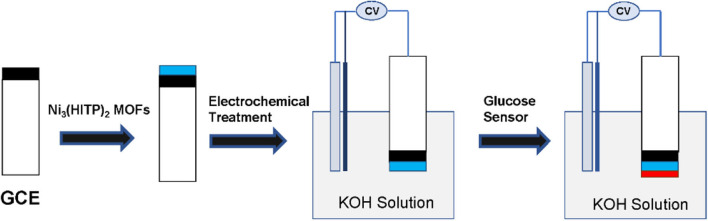
Schematic image of electrochemical activated Ni-MOFs electrode for glucose sensor.

The preparation of the Ni-MOFs glucose sensor was illustrated as Figure 1, following the sequence of the GCE polishing, Ni-MOFs synthesis, electrochemical activation, and glucose sensing. The polished GCE exhibited an even surface and excellent electronic transfer capacity, which is indicated by the cyclic voltammogram of K_4_Fe(CN)_6_ (Supplementary Figure 1). After that, the synthesized Ni_3_(HITP)_2_ MOFs mixed with Nafion was dropped on the polished GCE. Then, it was activated by the electrochemical method. The treated Ni-MOFs showed obvious reduction and oxidation peaks, which were sensitive to glucose and used as a non-enzymatic glucose sensor.

**Figure 2:**
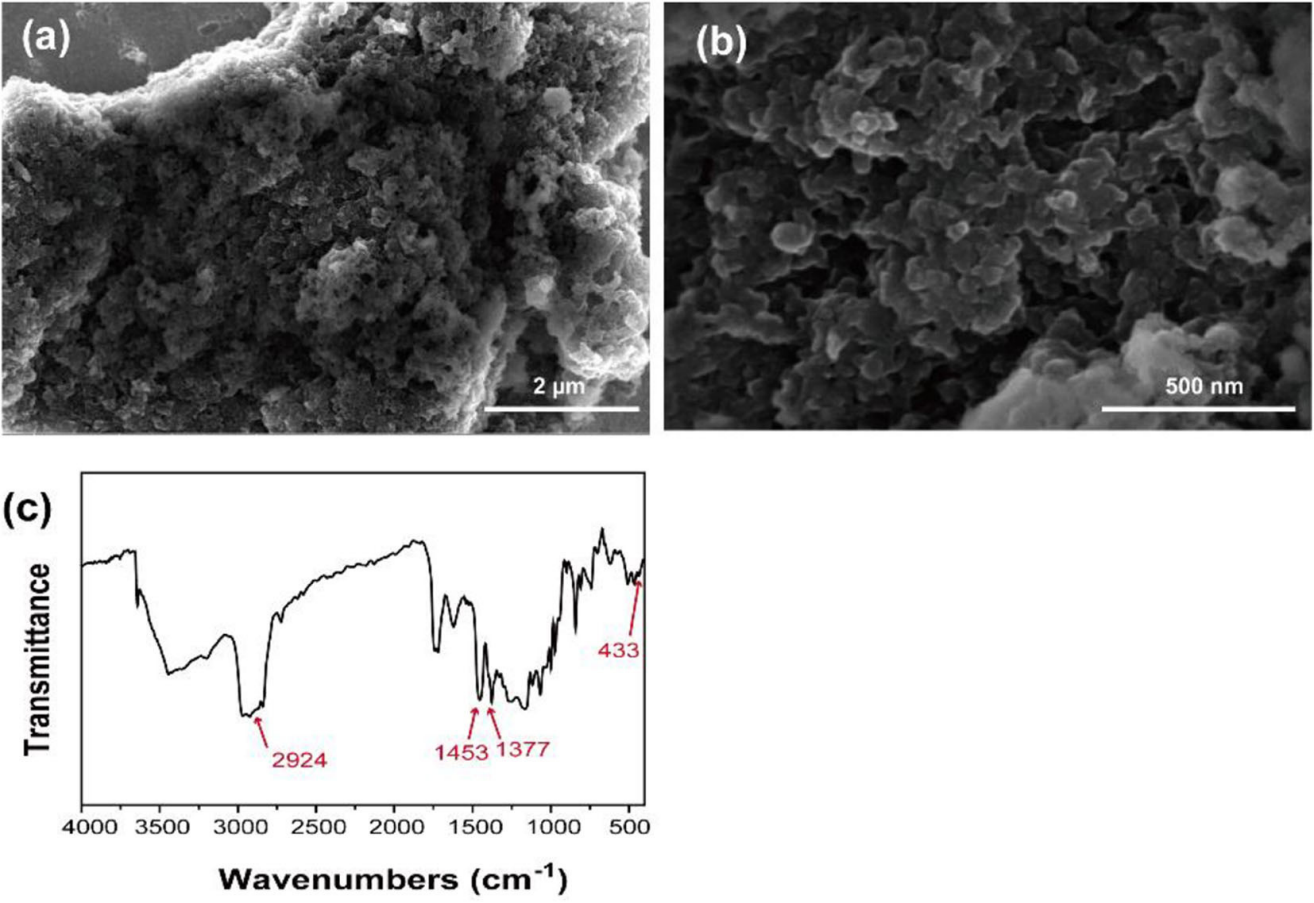
**(a,b)** SEM image with different magnification, **(c)** FTIR spectra of Ni-MOFs.

The prepared Ni-MOFs exhibited a rough surface with particle sizes around 50 nm in SEM image (Figure 2). In the FTIR spectra, the peak at 2924 cm^−1^ is the aromatic C-H stretching vibrations. The peak at 1453 and 1377 cm^−1^ was due to the C=C vibration of the aromatic polymer chain (Su et al., 2020). The peaks at 433 cm^−1^ were assigned to Ni-N stretching vibrations (Rocchiccioli-Deltcheff et al., 1978).

### Activation of Ni-MOFs Electrode

**Figure 3:**
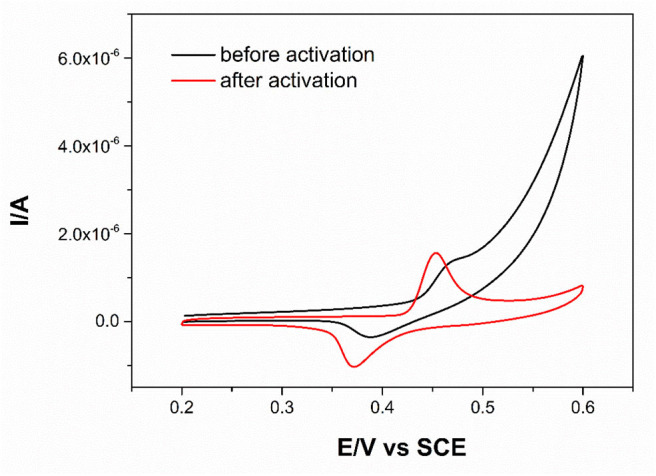
CV curves of Ni-MOFs before and after treatment in 0.1 M KOH, scan rate 50 mV s^−1^.

The electrochemical responses of MOFs before and after activation were studied (Figure 3). It can be seen that the voltammogram showed stronger anodic peaks or cathodic peaks after activation. Moreover, the anodic current and cathodic current became similar after activation. These results indicated that the activation could enhance the electrochemical reversibility. Two peaks appeared in the treated cyclic voltammogram, *i.e*., one in the anodic direction at 0.46 V and another in the cathodic direction at 0.37 V. These peaks are caused by the conversion of Ni(II)/Ni(III) to each other in the Ni-MOFs in the alkaline solution through the following reaction:

(1) $$\begin{array}{r} {\text{Ni}\left(\text{II}\right)}_{3}{\left(\text{HITP}\right)}_{2}⇌{\text{Ni}\left(\text{III}\right)}_{3}{\left(\text{HITP}\right)}_{2}+{\text{e}}^{-} \end{array}$$

The cyclic voltammogram of Ni-MOFs at different concentrations of KOH was also investigated (Supplementary Figure 2). Compared with other concentrations, the voltammogram in 0.1 M KOH showed stronger anodic and cathodic peaks. Therefore, KOH at the concentration of 0.1 M was chosen as the activation solution.

### Electrocatalytic Oxidation of Glucose

Cyclic voltammetry (CV) was used to study the glucose oxidation by treated Ni-MOFs. The CV curves showed that the peak current continually increased with the increasement of glucose concentration in the range of 0 to 10 mM with a linear calibration equation:

$$\begin{array}{l} I_{pa}=0.252\;C_{glucose}+2.076\left(R^{2}=0.954\right) \end{array}$$

**Figure 4:**
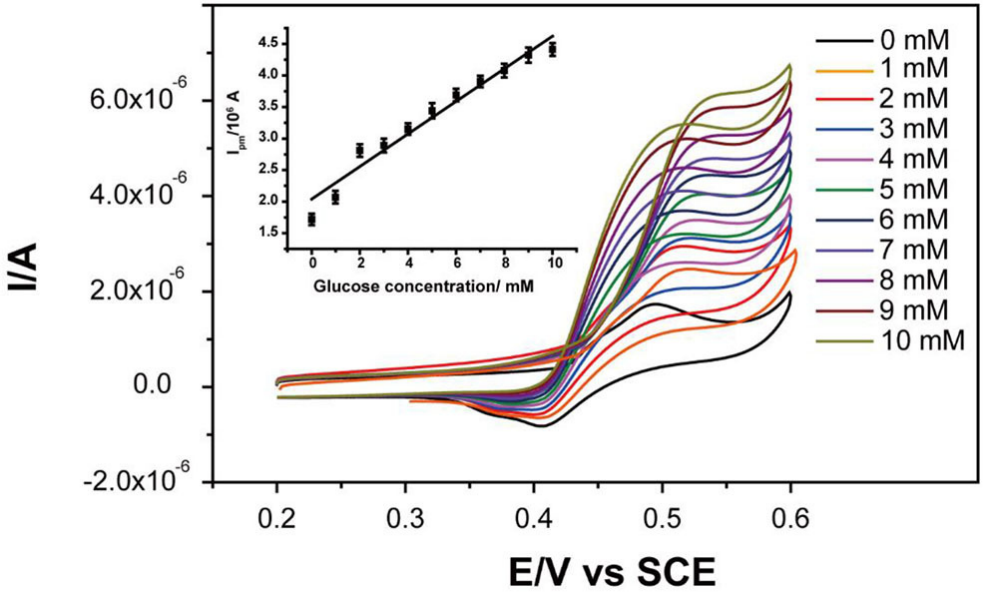
CV curves of Ni-MOFs in the absence and presence of different concentrations of glucose. Inset: the corresponding calibration curve of anodic peak current to glucose concentrations.

Where *I*_*pa*_ is the anodic peak current and *C*_*glucose*_ is the concentration of glucose (Figure 4).

This voltammogram indicated that Ni-MOFs could electro-catalyze the oxidation of glucose to gluconolactone *via* reaction 2, which was also in agreement with the nickel oxide-based glucose sensor (Abdel Hameed, 2013; Yang et al., 2013; Wang et al., 2015; Chelaghmia et al., 2018). It is known that diabetes mellitus is reflected by blood glucose concentrations higher or lower than the normal range of 4.4-6.6 mM (Chelaghmia et al., 2018). Therefore, the linear relationship in 1-10 mM could totally satisfy the requirement of continuous blood glucose monitoring.

(2) $$\begin{array}{l} {\text{Ni}\left(\text{III}\right)}_{3}{\left(\text{HITP}\right)}_{2}+\text{O}{\text{H}}^{-}+\text{glucose}⇌{\text{Ni}\left(\text{II}\right)}_{3}{\left(\text{HITP}\right)}_{2}\\ \;+\text{gluconolactone}+{\text{H}}_{2}\text{O}+{\text{e}}^{-} \end{array}$$

**Figure 5:**
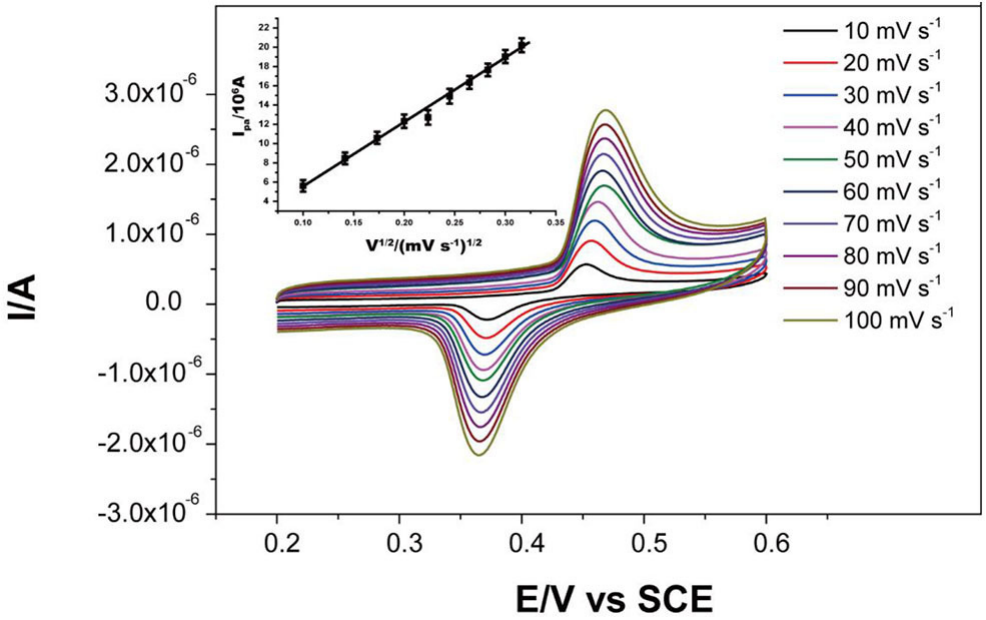
CV curves of Ni-MOFs in the presence of 2.0 mM glucose and 0.1 M KOH solution at scan rates from 10 to 100 mV s^−1^ at 25 °C. Inset: calibration curve of anodic peak current to square root of the scan rates (10-100 mV s^−1^).

The effects of scan rate were tested in the range of 10–100 mV s^−1^ in the presence of 2 mM glucose in 0.1 M KOH (Figure 5). The anodic peak current was proportional to the square root of the scan rate (v^1/2^), linearly following the linear regression equation:

$$\begin{array}{l} I_{pa}=6.631\times 10^{-7}v^{1/2}-1.117\left(R^{2}=\;0.992\right) \end{array}$$

This suggests the diffusion of the glucose to the electrode surface was the limiting step of the electrochemical reaction.

### The Selectivity and Stability of Ni-MOFs Electrode

**Figure 6:**
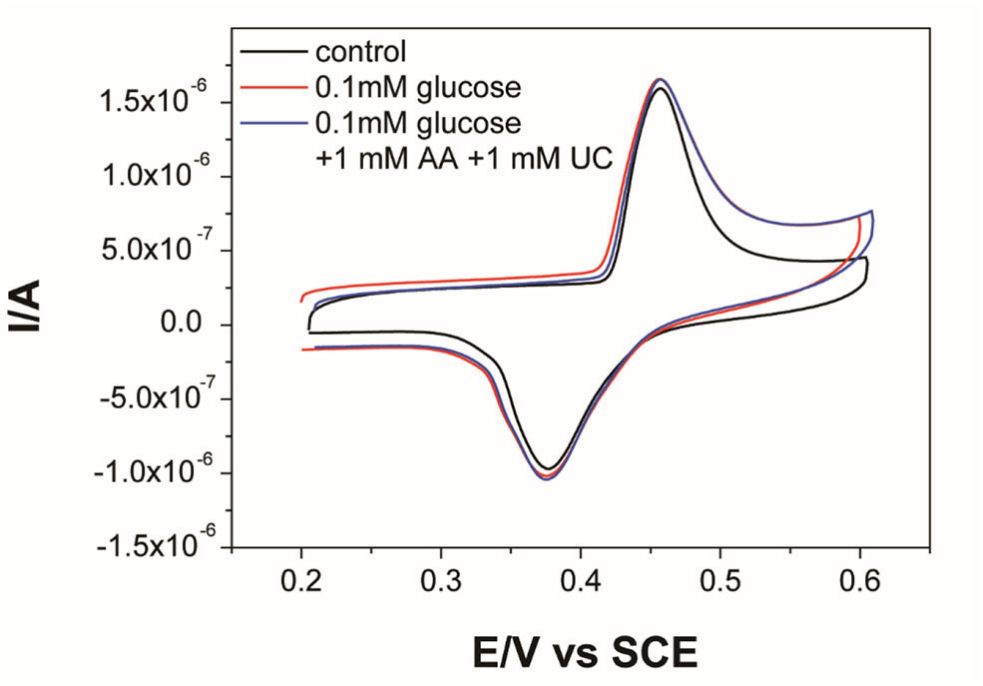
CV curves of Ni-MOFs electrode in 0.1 M KOH and 0.1 mM glucose, 1.0 mM ascorbic acid (AA), and 1.0 mM uric acid (UC).

The selectivity of the activated Ni-MOFs sensor was investigated in the presence of UA and AA, which are regarded as glucose interferents in human blood (Yuan et al., 2005). Even the concentration of glucose in normal human blood is higher than the interfering concentration; the 10-folds concentration of interference is persuasive. The cyclic voltammograms of MOFs sensor in the presence of glucose and 10-fold UA and AA were shown in Figure 6. Compared with 0.1 mM glucose, no significant difference was observed in the detection of 0.1 mM glucose in the mixture of 1 mM AA and 1 mM UC, indicating good selectivity for glucose monitoring. The long-term stability of the activated Ni-MOFs electrode has also been evaluated (Supplementary Figure 3). Through 7-day tests, there was no obvious difference in the cyclic voltammograms of electrodes in the alkaline solution, indicating good stability of the sensor. Furthermore, a comparative study of Ni_3_(HITP)_2_-based glucose sensor with the reported literature (Table 1) revealed that this MOFs exhibited a wider linear range and higher stability.

**Table 1:** Comparative characteristics of the non-enzymatic MOFs based glucose sensors.

**Active materials**	**Method**	**Potential (V)**	**Linear range (μM)**	**Stability**	**References**
Cu_2_(BTC)Cl(H_2_O)_4_	DPV	0.50	0.006–5000	92%	(Sun et al., 2018)
Ni-BTC	CV	0.55	5–3000; 3500–6000	–	(Chen et al., 2020)
Ni-BDC	CV	0.63	10–800	–	(Gumilar et al., 2020)
Ni/Co-TCPP	CV	0.40	1.0–3800	–	(Li et al., 2020a)
NiO/Cu-TCPP	CV	0.50	3–300	–	(Li et al., 2020a)
NiCo-MOFs nanosheets	CV	0.50	1–8000	–	(Li et al., 2019)
Ag/Co-MOFs	CV	0.55	5–550	30 times	(Liu et al., 2019)
Ni_3_(HITP)_2_	CV	0.50	0–10000	7 days	This work

* *DPV, differential pulse voltammetry; CV, cyclic voltammetry; BTC, benzenetricarboxylic acid; BDC, benzene dicarboxylic acid; TCPP, tetrakis(4-carboxyphenyl)phosphonium porphyrin; HITP, 2,3,6,7,10,11-hexaiminotriphenylene*.

## Conclusions

In conclusion, a non-enzymatic glucose sensor was developed through electrochemical activation of the Ni_3_(HITP)_2_ MOFs on GCE in an alkaline solution. Cyclic voltammetry technology was used to study the electrocatalytic oxidation of glucose on the surface of activated Ni-MOFs. The active MOFs displayed excellent electrocatalytic activity toward the glucose oxidation in 0.1 M KOH solution and showed a good linear relationship toward 0-10 mM. The diffusion of glucose to the electrode surface was the limiting speed for the electrochemical reaction. In addition, the MOFs sensor exhibited excellent selectivity toward glucose in the presence of AA and UC. Also, the electrode exhibited long-term stability.

The conventional fabrication, high performance, excellent stability, and low cost of the activated conductive Ni-MOFs suggests its suitability as a reliable non-enzymatic glucose sensor.

## Data Availability Statement

All datasets generated for this study are included in the article/ Supplementary Material.

## Author Contributions

YC: conceptualization, methodology, validation, formal analysis, data curation, and writing original draft. YT: methodology, validation, investigation, visualization, and writing-review and editing. PZ: methodology, resources, and visualization. LD: methodology, writing-review and editing, and visualization. WC: conceptualization, methodology, validation, data curation, visualization, and writing-review and editing. CW: conceptualization, methodology, resources, writing-review and editing, supervision, project administration, and funding acquisition. All authors contributed to the article and approved the submitted version.

## Conflict of Interest

The authors declare that the research was conducted in the absence of any commercial or financial relationships that could be construed as a potential conflict of interest.
