# Supplementary material for: Electrochemically Activated Conductive Ni-Based MOFs for Non-enzymatic Sensors Toward Long-Term Glucose Monitoring
Source: Front Chem. 2020 Nov 25;8:602752. doi: 10.3389/fchem.2020.602752 (PMC7723845; doi:10.3389/fchem.2020.602752)
Supplement: Supplementary file 1 [file Data_Sheet_1.docx]

Supplemental Documents

Electrochemical Activated Conductive Ni-based MOFs for Non-enzymatic Sensors Towards Long-term Glucose Monitoring

Yating Chen, Yulan Tian, Ping Zhu, Liping Du, Wei Chen*, Chunsheng Wu*

Institute of Medical Engineering, Department of Biophysics, School of Basic Medical Sciences, Health Science Center, Xi’an Jiaotong University, Xi’an, Shaanxi, 710061, China

* Correspondence: W. Chen (weiwcchen@xjtu.edu.cn) and C. Wu (e-mail:

wuchunsheng@xjtu.edu.cn)

Fig. S1 Cyclic voltammogram of polished GCE.

Fig. S2 Cyclic voltammogram of Ni-MOFs in different concentration of KOH.





Fig. S3 Cyclic voltammograms of Ni-MOFs electrode in 0.1 M KOH at different test times.
